# Antidepressant-induced reduction in betel-quid use in patients with depression: A pioneer clinical study

**DOI:** 10.1097/MD.0000000000018672

**Published:** 2020-01-03

**Authors:** Chung-Chieh Hung, Chien-Hung Lee, Chia-Min Chung, Srinivasan Nithiyanantham, Hsien-Yuan Lane, Ying-Chin Ko

**Affiliations:** a Graduate Institute of Clinical Medical Science, China Medical University; b Department of Psychiatry, China Medical University Hospital, Taichung; c Department of Public Health, Environmental Medicine Research Center, College of Health Sciences, Kaohsiung Medical University, Kaohsiung; d Environment-Omics-Disease Research Center, China Medical University Hospital, China Medical University; e Department of Psychology, College of Medical and Health Sciences, Asia University, Taichung, Taiwan.

**Keywords:** abstinence, addiction, antidepressant, betel-quid, depression

## Abstract

Betel-quid is commonly used around the world and is listed as a Group I carcinogen. Prior research has suggested a possible association between antidepressants and betel-quid use. We aimed to clarify the effects of antidepressant therapy in betel-quid chewers in the population of patients with depression.

We enrolled 204 patients with depressive disorders, collected their demographic information, and administered the Substance Use Severity Rating Scale for alcohol, cigarettes, and betel-quid and the Hamilton Depression Rating Scale. We compared betel-quid and non-betel-quid chewers and examined the effects of antidepressant therapy on betel-quid abstinence after previous exposure to betel-quid.

Patients with depression were reported a higher prevalence of 26% betel-quid chewing habits and patients who chewed betel-quid showed more severe depressive symptoms. After antidepressant therapy, the addictiveness of betel-quid was significantly reduced by 4 times.

This was a pioneering study showing that antidepressants could be a candidate for betel-quid cessation therapy. Future clinical trials are needed to verify their efficacy in reducing consumption for betel-quid addiction treatment.

## 1 Introduction

The Betel-quid (BQ) is a chewing mixture of dried or fresh ingredients from the areca catechu nut, with or without tobacco. Since 1985, its addictive properties have been reported by the International Agency for Research on Cancer.^[1]^ BQ has been listed as a Group 1 carcinogen in humans, either with or without tobacco.^[2,3]^

BQ chewing is a strong social bonding and cultural practice among individuals in SouthEast Asia, India, and South Pacific countries such as Papua New Guinea.^[4,5]^ An estimated 600 million people chew BQ worldwide,^[6]^ making it the fourth most popularly accepted psychoactive substance used in daily life.^[7]^

The prevalence of BQ use in the general population is around 10% worldwide.^[8]^ An investigation has revealed the current prevalence of BQ chewing to be 10.7% in men and 2.5% in women in Taiwan.^[9]^ Regarding symptoms of abuse in Taiwan, higher incidences of dependence (46.1%), craving (40.5%), and tolerance (27.1%) than average are reported.^[10]^ Based on research into worldwide BQ chewing from a study of six Asian countries, in specific groups, such as Hunan men (a province of China), Malaysian women, and the Indonesian and Nepalese populations, the incidence of BQ dependence even exceeds that of alcohol dependence.^[9]^ However, the severity of BQ dependence and the resulting psychiatric problems are rarely studied.

MAO-A catalyzes the deamination of biogenic amines in the blood or synapses and regulates the levels of dopamine, serotonin, norepinephrine, and catecholamine.^[11]^ Literature indicated the mechanisms affected by the active ingredients of BQ may interact with antidepressants. Areca nut regulates the expression of monoamine oxidase- A (MAO-A) Xp 11.3. In human study, our team has investigated that the MAO-A are associated with high exposure betel quid use.^[12]^ Besides, another literature has also detected that the ingredient of BQ stimulating the levels of monoamines (serotonin and dopamine) by the experimental design of MAO inhibition in rats.^[13]^

The duration of untreated depression (DUD) might be related with more severe depressive symptoms of the BQ chewers. Since they probably tended to had longer DUD because the first depressive episode under the condition of poor adherence and cooperation with medical treatment. The previous study shows that DUD is associated with the disability and outcome of the depressive disorders.^[14]^

Prior research suggests a possible association between antidepressant mechanisms and BQ.^[12]^ However, few studies have examined the relationship between BQ use and depressive disorders. The comparison of the contributing risks to depressive disorders between BQ chewers and non-BQ chewers is important for better understanding. The association between antidepressants and BQ dependence might indicate a potential future cessation treatment. Our study examined the association between severity of BQ use, depressive symptoms and the potency of BQ abstinence under antidepressant therapy in patients with depressive disorders.

## 2 Materials and methods

**Figure 1:**
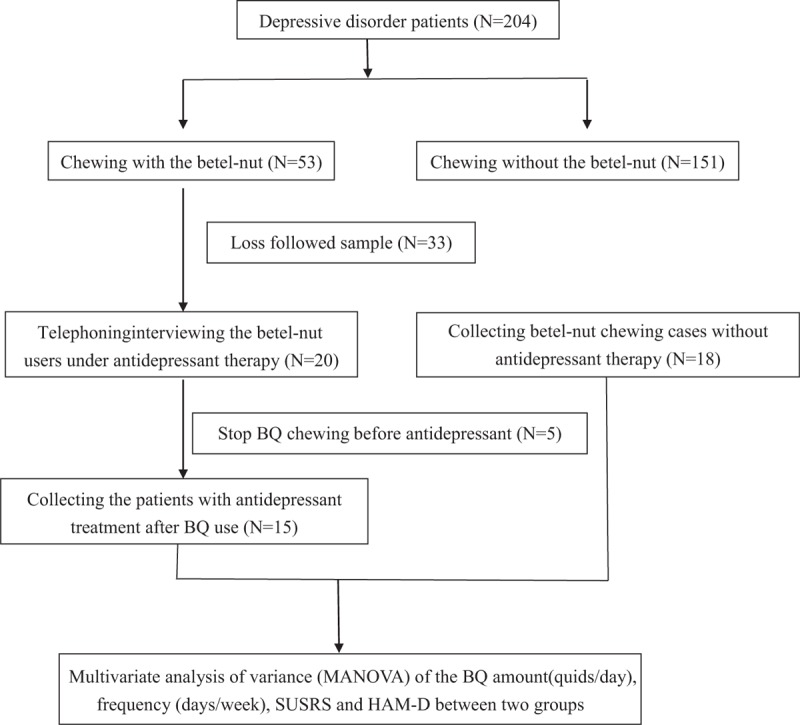
Flow diagram of the study design.

This study was approved by the China Medical University Hospital Institutional Review Board (IRB). All the participants gave written approval before the study. Our overall research flow diagram was shown in Figure 1. Participants were recruited from the psychiatric outpatient department of China Medical University Hospital in Taichung, Taiwan between August 2014 and August 2015. We applied DSM-5 (The Diagnostic and Statistical Manual of Mental Disorders Fifth edition) criteria^[15]^ to select patients with current depressive disorders. According to the DSM-5, this umbrella diagnosis includes major depressive disorder, persistent depressive disorder, disruptive mood dysregulation disorder, and other specified and unspecified depressive disorders. Substance/medication-induced depressive disorders were excluded to avoid confounds from other substances or medications.

### 2.1 Measures of demographic information and clinical characteristics

After the enrollment of eligible patients, we measured their Substance Use Severity Rating Scale (SUSRS) scores for alcohol, cigarettes, and BQ^[16]^ and their score on the Hamilton Depression Rating Scale (HAMD).^[17]^ Duration of antidepressant use was retrospectively traced and was defined as the duration of antidepressant dosing before this study. We included information on the use of all kinds of antidepressants. Those who had lost follow-up contact with the psychiatric service for at least 1 year were classified as zero for antidepressant use during this time.

We also collected data on the type of antidepressant used, including SSRIs (Selective Serotonin Reuptake Inhibitors), SNRIs (Serotonin Nor-epinephrine Reuptake Inhibitors), and NDRIs (Nor-epinephrine Dopamine Reuptake Inhibitors). Those that did not fall into one of the above three groups, such as tricyclic antidepressants, monoamine oxidase inhibitors, serotonin antagonists, reuptake inhibitors, and noradrenergic and specific serotonergic antidepressants, were classified as “others.”

### 2.2 Comparisons between BQ and non-BQ chewers among patients with depression

We divided the patients into 2 groups: BQ chewers and non-BQ chewers. The BQ group consisted of all patients with a current or former habit of BQ chewing. In the non-BQ group, the patients had never chewed BQ. We compared the groups on age, education, employment, and their SUSRS scores for alcohol, BQ, and cigarettes, and their HAMD scores.

### 2.3 Follow-up measures in BQ chewers before and after antidepressant treatment

A trained psychiatrist or psychologist made telephone contact with the patients to follow up after antidepressant treatment. Their level and frequency of BQ consumption, SUSRS score for BQ, and HAMD scores were collected as post antidepressant treatment measures.

### 2.4 Comparisons of the variables between antidepressant treatment and normal population without any intervention

We keep following up on the 20 patients who underwent the antidepressant treatment. We have collected another 18 subjects from the same hospital with BQ chewing habits without any antidepressant intervention in the past years. We obtained the information relating to the BQ chewing habits. The difference of their BQ chewing amount (quids/day), frequency (days/week), SUSRS and HAMD were compared.

### 2.5 Statistical analysis

The BQ and non-BQ chewing groups were compared on their clinical characteristics and associations. To carry out these analyses, the Student *t* test and Chi-squared test were employed. Multivariate analysis of variance (MANOVA) was applied to compare the intervention difference between the antidepressant treatment patients and natural BQ chewing cases.

## 3 Results

### 3.1 Demographic data and clinical characteristics of participants

We have collected the data from 204 patients with depressive disorders, in which the prevalence of BQ chewing was 26% (53 patients). The distributions of demographic and clinical characteristics in BQ chewing and non-BQ chewing groups are shown in Table 1. BQ chewing patients tended to be older (47 ± 11 years) and had higher SUSRS for alcohol (1.0 ± 2.7), cigarette (2.2 ± 2.8), and BQ (1.2 ± 2.7) consumption, and HAMD (13.2 ± 8.0), compared to their non-chewing counterparts (42 ± 14 years, 0.7 ± 0.6, 0.4 ± 1.4, 1.0 ± 2.1, and 10.3 ± 7.0) respectively.

**Table 1:**
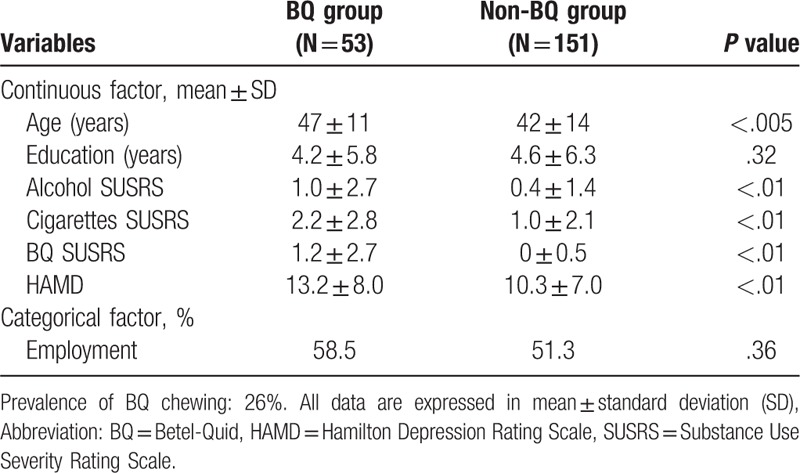
Demographic data and clinical features between BQ and non-BQ chewers among the patients with depressive disorders.

### 3.2 Characteristics and comparisons between successfully retained BQ-chewing participants and lost to follow-up

As shown in Table 2, the characteristics of successfully followed-up BQ-chewing patients were as follows: age, 46 ± 10 years; SUSRS-alcohol, 0.8 ± 1.8; SUSRS-cigarettes, 1.9 ± 2.4; SUSRS-BQ, 1.3 ± 3.6; HAMD, 15.5 ± 7.7; and duration of antidepressant treatment, 7.1 ± 6.7 years than the patients were lost to follow-up (24 ± 10 and 15.5 ± 11.3 years, respectively). There is no significant difference between the retained and lost BQ chewers of 6 variables.

**Table 2:**
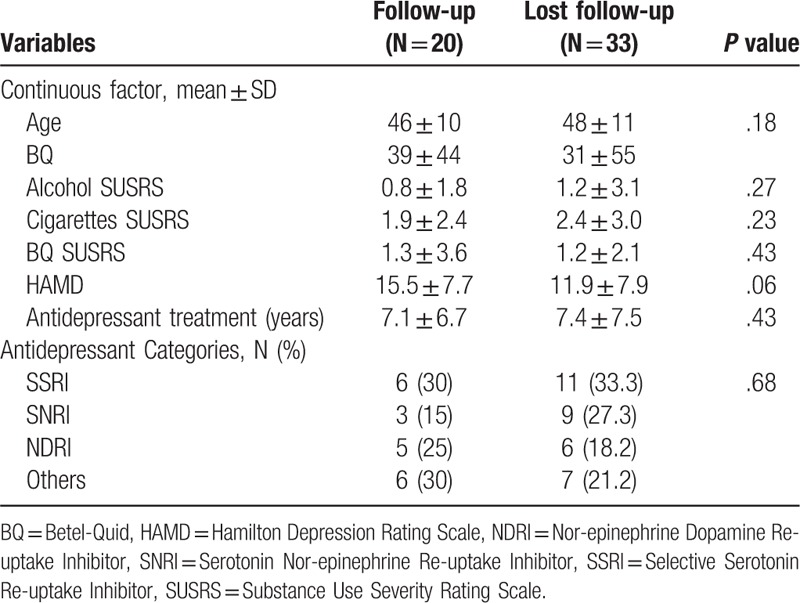
Comparisons of the difference between the follow-up and lost follow-up BQ chewers.

The successfully retained BQ chewers were treated with antidepressants in the following proportions: SSRIs, 30%; SNRIs, 15%; NDRIs, 25%; and others, 30%. Their counterparts who could not be followed up were treated in the following proportions: SSRIs, 33.3%; SNRIs, 27.3%; NDRIs, 18.2%; and others, 21.2%. There is no significant difference in the category of antidepressant used between the 2 groups.

### 3.3 Differences in BQ use and HAMD scores before and after antidepressant treatment

We followed up with 20 patients from the BQ-chewing group (response rate: 40%). After antidepressant therapy, we found that the mean level of BQ use in this group fell from 39 ± 43 to 4 ± 6 quids/day, the frequency of BQ consumption fell from 5.3 ± 3 to 0.7 ± 1.1 days/week, SUSRS scores for BQ fell from 1.3 ± 3.6 to 0.3 ± 0.8, and HAMD scores fell from 15.5 ± 7.7 to 2.4 ± 2.5 (Table 3). We found significant differences in all 4 variables before and after the antidepressant intervention.

**Table 3:**
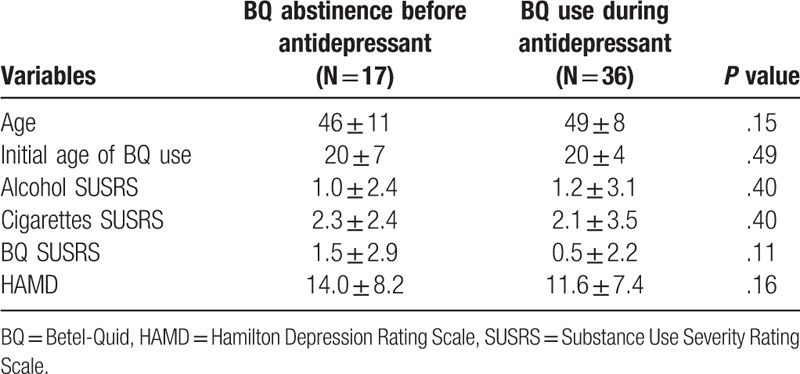
Comparisons between the groups with antidepressant before the abstinence of BQ (N = 17) and continuous intervention (N = 36).

### 3.4 Comparisons of the variables between antidepressant treatment and normal population without any intervention

The 20 depressive patients with BQ chewing followed for the consecutive 2 years and collected another 18 participants with mild anxious or depressive symptoms who did not receive any antidepressant intervention. The results were demonstrated in Table 4. We have calculated the difference of BQ consuming amount (quids/ day), chewing frequency (days/week), SUSRS (BQ) and HAMD before and after the observation period. We found that the difference of intervention group BQ chewing amount 39.2 quids/day, frequency 4.8 days/week, SUSRS (BQ) 2.3, and HAMD 11.8 while the counterpart was revealed as the difference of BQ chewing amount 2.6 quids/day, frequency 0.4, SUSRS (BQ) 0.1, and HAMD 1.3. Statistical significance existed in the interaction *P* value in the reduction of BQ consuming amount, frequency, SUSRS and HAMD.

**Table 4:**
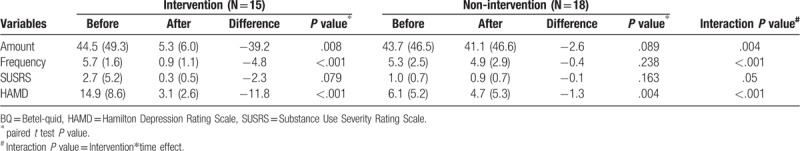
Difference in BQ use characteristics, dependent severity and HAMD score before and after antidepressant treatment between BQ chewing patients with and without the antidepressant intervention (N = 15, 18).

## 4 Discussion

We observed a BQ chewing prevalence of 26% in male patients diagnosed with depressive disorders according to the DSM-5. This was higher than the average prevalence in Taiwan and even worldwide.^[4,8]^ Comparisons between the BQ chewing and non-BQ chewing patient groups showed statistically significant differences in several variables: age; SUSRS scores for alcohol, cigarettes, and BQ; and HAMD scores. Of these results, the novel findings from our study revealed that the depressive patients chewed BQ suffered from more severe depression according to the HAMD scores.

We found that antidepressant treatment reduced the clinical severity of BQ use, as measured by the total amount consumed, frequency of consumption, and SUSRS (BQ) scores. Depressive symptoms, as rated by the HAMD, were also significantly reduced. This finding supports our previous reports suggested an association between antidepressants and a reduction in the occurrence of oral cancers, implicating in the effect a decrease in the amount and severity of BQ use.^[18]^ Such results might suggest that patients with depression perhaps use BQ as a form of self-medication for their depressive disorders, and thus do not need to continue using it when their depression is treated. The active ingredients of BQ, arecoline and arecaidine, are plant alkaloids that readily cross the human blood-brain barrier.^[19]^ The BQ metabolites from the influential role from the animal cortical neurotransmitter dopamine, a kind of MAO inhibition such as the traditional pathway of antidepressant might contribute to the temporal effects between BQ and antidepressant.^[20]^ However, self-medication does not seem most likely explanation for elevated BQ use in patients with depression: Because of poor adherence to treatment or poor response to antidepressants, such patients’ depressive symptoms were actually more severe than those of the non-BQ chewers.

Counseling or other rehabilitation programs have been revealed to have poor efficacy in BQ dependence therapy,^[21]^ and chewers who want or intend to quit always do not have definite plans for abstinence.^[22]^ There are no pharmacologically based replacement therapies for BQ abuse, and the addictive properties of BQ limit the effectiveness of counseling-based quitting programs.^[23]^ BQ dependence is highly represented with the symptoms of tolerance and withdrawal to become the current chewer. In addition, BQ craving plays a role in the continuation and development of addiction. The majority of the Asian users of BQ already have BQ use disorder (BUD), which is correlated with the risk of OPMD. Hence, we required an immediate psychiatric management plan for users of BQ.^[24]^

Our current prospective and observational study of the associations between depression and BQ chewing, and meanwhile between anti-depressant treatment for depression and BQ, based on 204 patients with depressive disorders at a single outpatient clinic potentially offered useful information on risk factors for betel-quid use. It was indicated a possible pharmacological treatment in the future.

Despite its novel implications, our study suffered from limitations resulting from the cross-sectional design, including the difficulty of establishing causal relationships using such a design. In the follow-up contact by telephone, poor motivation in the participants might have contributed to recall bias. The high drop-out rate (33 of the 53 initial cohort patients chewing BQ) deserved alert of the substantial risk of bias, though the drop-out and the retained group patients did not differ significantly in the 6 measured variables (Table 2). The high drop-out rate also described the nature of the poor motivation of the study cohort. This might also contribute to useless counseling and psychotherapy for the abstinence of BQ addiction. The fact that the available patients with a clinical depression diagnosis had already undergone continuous antidepressant therapy may also have influenced our findings.

## 5 Conclusion

Patients with depressive disorders had an elevated prevalence of BQ chewing (26%). The present study was a first step towards understanding a possible correlation between the severity of depressive symptoms, signs and substance use of BQ in a clinical setting. Antidepressant treatment reduced the clinical severity of BQ use, as measured by the amount and frequency of consumption and SUSRS (BQ) scores. Depressive symptoms, as measured by HAMD scores, were also significantly reduced. A more powerful study design, such as a randomized clinical trial, may in the future allow verification of the relationships between BQ use, depressive symptoms, and antidepressant use. The novel findings of our study were antidepressant might be the therapeutic agent for the BQ addiction patients, mainly via the MAO-A pathway. This study provides preliminary data and requires replication in larger trials.

Srinivasan Nithiyanantham orcid: 0000-0001-9217-1269.
